# Rapid decline of cerebrospinal fluid biomarkers of axonal injury and neuroinflammation after initiation of antiretroviral therapy in HIV

**DOI:** 10.1007/s13365-025-01287-2

**Published:** 2025-10-21

**Authors:** Linn Renborg, Aylin Yilmaz, Staffan Nilsson, Henrik Zetterberg, Kaj Blennow, Magnus Gisslén

**Affiliations:** 1 Department of Infectious Diseases, Institute of Biomedicine, Sahlgrenska Academy, University of Gothenburg, Gothenburg, Sweden; 2 Department of Infectious Diseases, Sahlgrenska University Hospital, Region Västra Götaland, Gothenburg, Sweden; 3 Department of Laboratory Medicine, Institute of Biomedicine, Sahlgrenska Academy, University of Gothenburg, Gothenburg, Sweden; 4 Department of Psychiatry and Neurochemistry, Institute of Neuroscience and Physiology, University of Gothenburg, Mölndal, Sweden; 5 Clinical Neurochemistry Laboratory, Sahlgrenska University Hospital, Mölndal, Sweden; 6 Department of Neurodegenerative Diseases, UCL Institute of Neurology, Queen Square, London, UK; 7 UK Dementia Research Institute at UCL, London, UK; 8 Hong Kong Center for Neurodegenerative Diseases, Clear Water Bay, Hong Kong China; 9 Wisconsin Alzheimer’s Disease Research Center, School of Medicine and Public Health, University of Wisconsin, University of Wisconsin-Madison, Madison, WI USA; 10 Pitié-Salpêtrière Hospital, Paris Brain Institute, ICM, Sorbonne University, Paris, France; 11 Neurodegenerative Disorder Research Center, Division of Life Sciences and Medicine, Department of Neurology, Institute on Aging and Brain Disorders, University of Science and Technology of China and First Affiliated Hospital of USTC, Hefei, P.R. China

**Keywords:** HIV associated brain injury, Neurofilament light (NfL), Cerebrospinal fluid biomarkers, Neuroinflammation, Antiretroviral therapy (ART)

## Abstract

Persistent intrathecal immune activation and neuronal injury remain common in people with HIV (PWH) despite effective antiretroviral therapy (ART). We examined longitudinal trajectories of cerebrospinal fluid (CSF) neurofilament light (NfL), a marker of axonal injury, together with neuroinflammatory biomarkers following ART initiation. Ninety-nine PWH from the Gothenburg HIV CSF Study Cohort who achieved viral suppression were included, with CSF samples collected before and after treatment initiation. NfL and a panel of biomarkers including YKL-40, sTREM-2, neopterin, and GFAP were analyzed. CSF NfL declined rapidly, from a mean of 673 ng/L at baseline to 592 ng/L after three months and 490 ng/L after twelve months. All inflammatory biomarkers showed parallel and significant decreases. Prior to ART, 25% of participants had elevated NfL levels; this subgroup displayed higher baseline inflammation, and the steepest biomarker declines after treatment initiation. In participants with normal baseline NfL, inflammatory markers decreased while NfL remained stable. Beyond one year, no further reductions were evident. These longitudinal findings demonstrate that ART rapidly and effectively reduces CSF biomarkers of neuronal injury and neuroinflammation in HIV, with the greatest benefit in individuals with baseline axonal damage.

## Introduction

The central nervous system (CNS) is infected by HIV within weeks of transmission, establishing a persistent viral reservoir that drives chronic intrathecal immune activation. Without treatment, this process can lead to neuronal injury (Gisslén et al. 1994; Zayyad and Spudich 2015). The clinical manifestations of HIV associated brain injury range from subtle neurocognitive deficits to severe HIV-associated dementia (HAD), historically associated with high morbidity and mortality (Antinori et al. 2007; Nightingale et al. 2023). Since the introduction of effective antiretroviral treatment (ART), the incidence of HAD has fallen dramatically (Lescure et al. 2011; Chan and Brew 2014; Sacktor et al. 2016). Nevertheless, an increased prevalence of neurocognitive complaints has frequently been reported in people with HIV (PWH) despite viral suppression, but the extent to which these are due to ongoing neuronal injury, comorbidities such as cardiovascular risk factors, or residual damage from previous injury remains unclear (Booiman et al. 2017; Robertson et al. 2021).

A range of cerebrospinal fluid (CSF) biomarkers can be used to assess intrathecal immune activation. Neopterin, produced by activated macrophages and microglia (Fuchs et al. 1988), is elevated at all stages of HIV infection. Although levels decrease after ART initiation, they remain elevated in up to 50% of PWH on effective therapy (Yilmaz et al. 2013; Ulfhammer et al. 2018). YKL-40 (chitinase-3-like protein 1, CHI3L1), a marker of astrocytic activation in neuroinflammatory disorders, is similarly increased in chronic HIV (Burman et al. 2016; Baldacci et al. 2017; Hermansson et al. 2019). Initiation of ART reduces CSF neopterin (Hellmuth et al. 2019) and YKL-40 (Peluso et al. 2017) levels in acute but not chronic HIV, indicating a possibility to prevent some degree of neuroinflammation by early initiation of ART. Soluble triggering receptor expressed on myeloid cells 2 (sTREM2) is an immune receptor glycoprotein expressed by myeloid-lineage cells. In the CNS, sTREM2 is produced by activated microglia and macrophages (Neumann and Takahashi 2007), and its expression increases with ageing, in neurodegenerative conditions such as Alzheimer’s disease and multiple sclerosis (Piccio et al. 2008; Mattsson et al. 2017), and in advanced HIV (Gisslén et al. 2019). Glial fibrillary acidic protein (GFAP) is the major structural protein in astrocytes and elevated CSF levels are associated with astrocytic injury or astrocytosis, as observed in other viral CNS infections (Grahn et al. 2013; Veje et al. 2023), CNS trauma, and inflammatory diseases (Fukuyama et al. 2001; Axelsson et al. 2011; Neselius et al. 2012). However, findings in the context of HIV have been inconsistent, and neither HIV-infection without dementia, nor HAD, has been consistently linked to elevated GFAP levels (Sporer et al. 2004).

The light subunit of the neurofilament protein (NfL) in CSF is a sensitive marker of axonal injury and neurocognitive impairment in HIV (Hagberg et al. 2000; Gisslén et al. 2007; Abdulle et al. 2007) as well as several other infectious, neuroinflammatory and neurodegenerative conditions, such as viral encephalitis, COVID-19, multiple sclerosis, amyotrophic lateral sclerosis, and Huntington’s disease (Grahn et al. 2013; Khalil et al. 2018; Bridel et al. 2019; Westman et al. 2021; Veje et al. 2023). In HIV, CSF NfL levels are highest in individuals with HAD or opportunistic infections, but elevated concentrations can also be found in asymptomatic individuals, particularly with low CD4 levels (Yilmaz et al. 2017). ART initiation leads to a marked reduction in CSF NfL levels, though they often remain slightly higher than in HIV-negative controls (Mellgren et al. 2007; Krut et al. 2014; Van Zoest et al. 2018; Anderson et al. 2020). Whether this residual elevation reflects ongoing neuronal injury, incomplete recovery after treatment initiation, or delayed normalization is not yet established. CSF levels of the glial markers neopterin and sTREM2, and the astroglial marker YKL-40, correlate with CSF NfL, supporting the role of neuroinflammation in axonal injury during HIV infection (Hagberg et al. 2000; Hermansson et al. 2019; Gisslén et al. 2019; Gisslen et al. 2021).

The main objective of this study was to characterize the longitudinal trajectory of CSF NfL following ART initiation in chronic HIV infection. Secondary objectives were to evaluate changes in CSF YKL-40, sTREM2, GFAP, and neopterin, and to assess their correlations with NfL over time.

## Methods

### Participants

In this retrospective, longitudinal study we included participants with chronic HIV from the Gothenburg HIV CSF Study cohort who initiated ART between 1997 and 2019 (Hagberg and Gisslén 2023). To be eligible, participants were required to have an archived CSF sample obtained 0–50 days prior to ART initiation and follow-up CSF samples for at least 12 months after treatment start. All participants achieved plasma HIV RNA < 50 copies/ml within one year from ART initiation. Individuals with secondary virological failure (plasma HIV RNA > 200 copies/ml in two consecutive samples or > 500 copies/mL in one sample) and those with CNS opportunistic infections or other CNS complications, such as traumatic injury or stroke were excluded. Data from participants with treatment interruption or virological failure were included only up to the time of failure. All available CSF samples from inclusion until the end of follow up in December 2020 were included in the analysis.

### Laboratory methods

CSF NfL concentration was measured using a sandwich ELISA method (NF-light^®^ ELISA kit, UmanDiagnostics AB, Umeå, Sweden) (Petzold et al. 2010) with a lower limit of detection of 50 ng/L. Neopterin concentration was determined using a commercially available immunoassay (BRAHMS, Berlin, Germany), with an upper normal limit in CSF of 5.8 mmol/L (Hagberg et al. 1993). CSF YKL-40 concentration was measured using a commercially available sandwich immunoassay with electrochemiluminescence detection (Meso Scale Discovery, Rockville, MD, USA), no reference limits established. CSF sTREM2 concentration was determined using an in-house immunoassay with electrochemiluminescence detection, as described previously in (Banerjee et al. 2020), with no established reference limits. CSF GFAP concentration was measured using an in-house ELISA, as previously described (Rosengren et al. 1994), with upper reference limits of 750 ng/mL (20–60 years) or 1250 ng/mL (> 60 years). HIV RNA in blood and CSF was measured using the Cobas TaqMan assay version 1 or 2 (Roche Diagnostic Systems, Hoffmann-La Roche, Basel, Switzerland), with lower quantification limits of 50 and 20 copies/ml, respectively. Other measurements were performed using standard clinical chemistry assays in the local clinical laboratory.

### Statistical analysis

Where applicable, NfL values were age adjusted to 50 years for longitudinal analysis using a previously described formula (Yilmaz et al. 2017), with an upper normal limit (adjusted to 50 years of age) of 967 ng/L. Continuous variables, except age, were log_10_ transformed before analysis. To account for variation in time between samples, interpolation was used to estimate log concentrations at 3 and 12 months, which were then back-transformed to geometric means. All presented means are geometric means, referred to simply as “mean”. The average decline from baseline to 3 and 12 months was evaluated using paired-sample t-test and expressed as percent decline per month. Between-group comparisons were performed using independent t-test. Correlations of log-transformed biomarker concentrations were calculated using partial correlations adjusted for age. Linear mixed-effect models were applied to evaluate long-term biomarker kinetics beyond one year of follow-up. All analyses were performed using IBM SPSS Statistics, version 29, and R, version 4.2.2.

### Ethics approval and consent to participate

All participants gave their informed consent, and the study was approved by the Reginal Ethics Review Board in Gothenburg, Sweden (Ö588-01), and performed in accordance with the Helsinki Declaration.

## Results

Ninety-nine participants were included in the study, with a median follow-up of 3.8 years and a total of 621 CSF samples. Thirty-four participants were assigned female at birth, and the median age was 40 years. Median HIV RNA levels in plasma and CSF were 4.99 log_10_ and 3.80 log_10_, respectively, and the median CD4^+^ T-cell count was 260 cells/µL (Table 1). The initial treatment regimen included two nucleoside reverse transcriptase inhibitors combined with a boosted protease inhibitor in 39 participants, an integrase inhibitor in 32, and a non-nucleoside reverse transcriptase inhibitor in 25.

**Table 1:** Baseline characteristics of participants; total cohort and divided according to baseline age-adjusted NfL level: above (High) or below (Normal) upper normal limit. Between-group comparisons performed using independent t-test

Characteristic	All (*n* = 99)	High NfL (*n* = 25)	Normal NfL (*n* = 74)
Female at birth	34 (34%)	9 (36%)	25 (34%)
	Median (IQR)		
Age	40 (34–51)	38 (35–49)	41 (32–51)
CD4^+^ T-cell count (cells/µL)	260 (120–410)	150 (30–260) *	300 (150–460) *
Plasma HIV RNA (log_10_ copies/mL)	4.99 (4.36–5.47)	5.39 (5.04–5.93) *	4.65 (4.23–5.38) *
CSF HIV RNA (log_10_ copies/mL)	3.89 (3.20–4.53)	4.00 (2.57–4.72)	3.88 (3.30–4.50)
Follow up (years)	3.8 (2.1–8.8)	4.4 (2.2–11.7)	3.7 (2.0–8.0)
Samples/participant	5 (4–8)	5 (4–12)	4 (3–7)

IQR = Interquartile range, CSF = Cerebrospinal fluid, * indicates significant difference between NfL groups

**Fig. 1:**
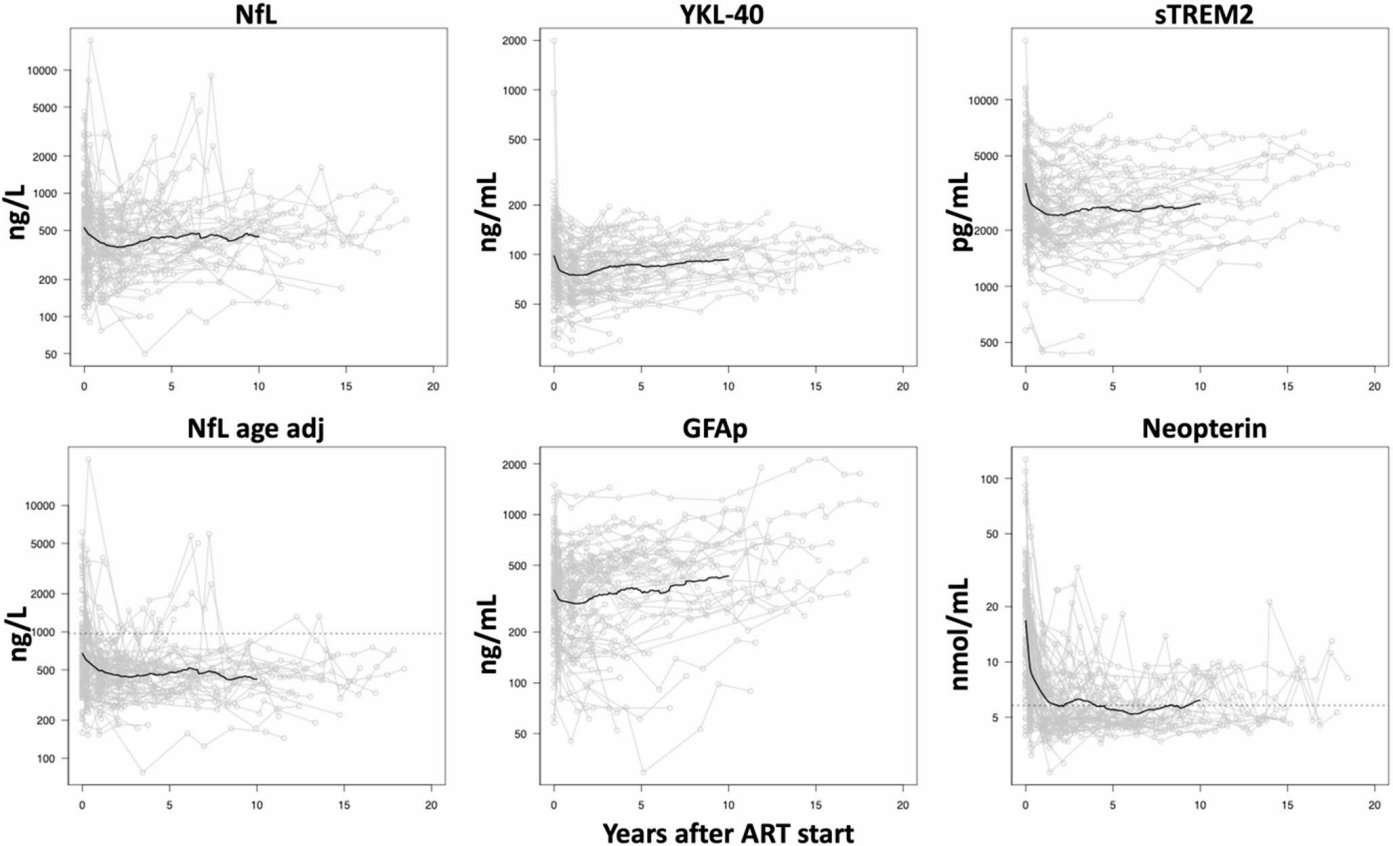
Spaghetti plots of longitudinal follow-up of cerebrospinal fluid biomarkers. Black line indicates mean values. Stippled line in age adjusted NfL (adjusted to 50 years) and in neopterin indicates upper normal limit (967 ng/L and 5.8 nmol/mL, respectively

At baseline, mean NfL levels, adjusted to 50 years, were 673 ng/L. Twenty-five participants (26%) had NfL levels above normal reference values (age adjusted mean NfL 1,983 ng/L compared to 467 ng/L in those with normal baseline levels). Participants with elevated NfL had significantly higher plasma HIV RNA (median 5.38 log_10_ versus 4.65 log_10_) and lower CD4^+^ T-cell counts (164 cells/µL versus 295 cells/µL), as well as significantly higher CSF levels of all inflammatory biomarkers, while no difference was found in CSF HIV RNA between NfL groups. There were no significant differences between treatment regimens in baseline or follow-up NfL levels at any timepoint, nor in NfL changes. Similarly, no significant differences were observed depending on sex assigned at birth. The mean levels of astrocyte markers YKL-40 and GFAP were 98 ng/mL and 356 pg/mL, respectively. Mean level of microglial markers neopterin and sTREM2 were 16.8 ng/mL and 3,540 pg/mL, respectively. All inflammatory biomarkers were significantly higher at baseline in the subgroup with elevated NfL. Table 2; Fig. 1.

**Table 2:** Cerebrospinal fluid biomarker levels at baseline, 3 months and 12 months, estimated through interpolation, in total cohort (”All”, *n* = 99) and subgrouped according to baseline NfL levels (”High”, *n* = 25 or ”Normal”, *n* = 74), analyzed using paired-sample t-test

Biomarker	BL NfL	BL	3 months	12 months
		Geometric mean (95% CI)		
NfL ng/L	all	523 (440–623)	464 (395–546)	395 (345–452)
	high	1542 (1201–1981)	880 (655–1183)	532 (391–722)
	normal	363 (316–418)	374 (316–443)	357 (308–413)
NfL (age adj) ng/L	all	673 (576–785)	592 (517–679)	490 (438–547)
	high	1983 (1548–2539)	1123 (849–1484)	662 (497–883)
	normal	467 (426–512)	477 (421–540)	442 (397–492)
YKL-40 ng/mL	all	98 (87–111)	83 (76–90)	75 (70–81)
	high	150 (106–212) *	99 (84–116) *	77 (68–88)
	normal	86 (77–96) *	78 (71–86) *	75 (68–82)
GFAP ng/mL	all	356 (312–406)	316 (281–355)	299 (264–340)
	high	476 (380–596) *	353 (280–444)	320 (248–413)
	normal	324 (278–378) *	305 (266–350)	293 (252–340)
sTREM2 pg/mL	all	3552 (3160–3993)	2851 (2576–3156)	2494 (2254–2758)
	high	5502 (4436–6823) *	3478 (2903–4167) *	2790 (2329–3342) *
	normal	3083 (2725–3487) *	2676 (2376–3015) *	2402 (2128–2712) *
Neopterin nmol/mL	all	16,8 (14,5–19,5)	9,2 (8,2–10,4)	6,6 (6,1–7,2)
	high	26,2 (19,5–35,2) *	10,3 (7,9–13,4)	6,4 (5,3–7,7)
	normal	14,5 (12,3–17,0) *	8,9 (7,8–10,1)	6,7 (6,1–7,3)

BL: baseline. NfL age adj: age adjusted to 50 years

* Significant difference of inflammatory biomarker levels between NfL subgroups

NfL and all inflammatory biomarkers decreased significantly at both 3 and 12 months in the entire cohort, as well as in those with elevated baseline NfL. In participants with normal NfL levels, neopterin, YKL-40, and sTREM2 were significantly lower after both 3 and 12 months, while NfL and GFAP did not decrease significantly at any timepoint. The average decline of age-adjusted NfL during the first three months was 4.3% per month in the entire cohort and 20.9% per month in the subgroup with elevated NfL at baseline. In the subgroup with normal NfL at baseline, there was a small increase of 0.7% per month. From baseline to 12 months, the average monthly decline was 2.7% for all, 9.6% in the NfL-“high” group and 0.5% in the NfL-“normal” group. A similar pattern of decline was observed for the inflammatory biomarkers, with significantly larger monthly decreases from baseline to 3 months in the subgroup with elevated baseline NfL levels. All decline rates are presented in Table 3.

**Table 3:** Average monthly decline of cerebrospinal biomarkers from baseline to 3 and 12 months of follow-up, in total cohort (”All”, *n* = 99) and subgrouped according to baseline NfL levels (”High”, *n* = 25 or ”Normal”, *n* = 74)

Biomarker		Decline/month 0–3		Decline/month 0–12	
		% (95% CI)	p	% (95% CI)	p
NfL ng/L	all	4.0 (0.1–8.1)	0.044	2.5 (1.3–3.7)	< 0.001
	high	20.6 (9.8–32.4)	< 0.001	9.3 (6.5–12.2)	< 0.001
	normal	–1.0 (–4.4–2.6)	0.573	0.2 (–0.6–1.1)	0.586
NfL (age adj) ng/L	all	4.3 (0.4–8.4)	0.031	2.7 (1.5–3.9)	< 0.001
	high	20.9 (10.1–32.7)	< 0.001	9.6 (6.7–12.5)	< 0.001
	normal	–0.7 (–4.2–2.8)	0.682	0.5 (–0.3–1.3)	0.242
YKL-40 ng/mL	all	5.6 (2.8–8.5)	< 0.001	2.2 (1.3–3.1)	< 0.001
	high	14.9 (4.0–26.9)	0.009	5.6 (2.2–9.1)	0.002
	normal	2.7 (1.4–3.9)	< 0.001	1.1 (0.7–1.4)	< 0.001
GFAP ng/mL	all	3.7 (0.9–6.5)	0.009	1.3 (0.5–2.2)	0.001
	high	10.5 (3.1–18.3)	0.007	3.3 (1.0–5.6)	0.007
	normal	1.5 (–1.2–4.3)	0.270	0.7 (–0.1–1.5)	0.068
sTREM2 pg/mL	all	7.8 (5.7–9.9)	< 0.001	3.0 (2.4–3.7)	< 0.001
	high	16.5 (10.3–23.1)	< 0.001	5.8 (3.9–7.7)	< 0.001
	normal	5.1 (3.4–6.7)	< 0.001	2.1 (1.6–2.6)	< 0.001
Neopterin nmol/mL	all	22.0 (17.4–26.9)	< 0.001	8.1 (6.9–9.4)	< 0.001
	high	36.7 (25.1–49.5)	< 0.001	12.4 (9.7–15.3)	< 0.001
	normal	17.4 (12.8–22.2)	< 0.001	6.7 (5.4–8.0)	< 0.001

NfL (age adj): age adjusted to 50 years

**Fig. 2:**
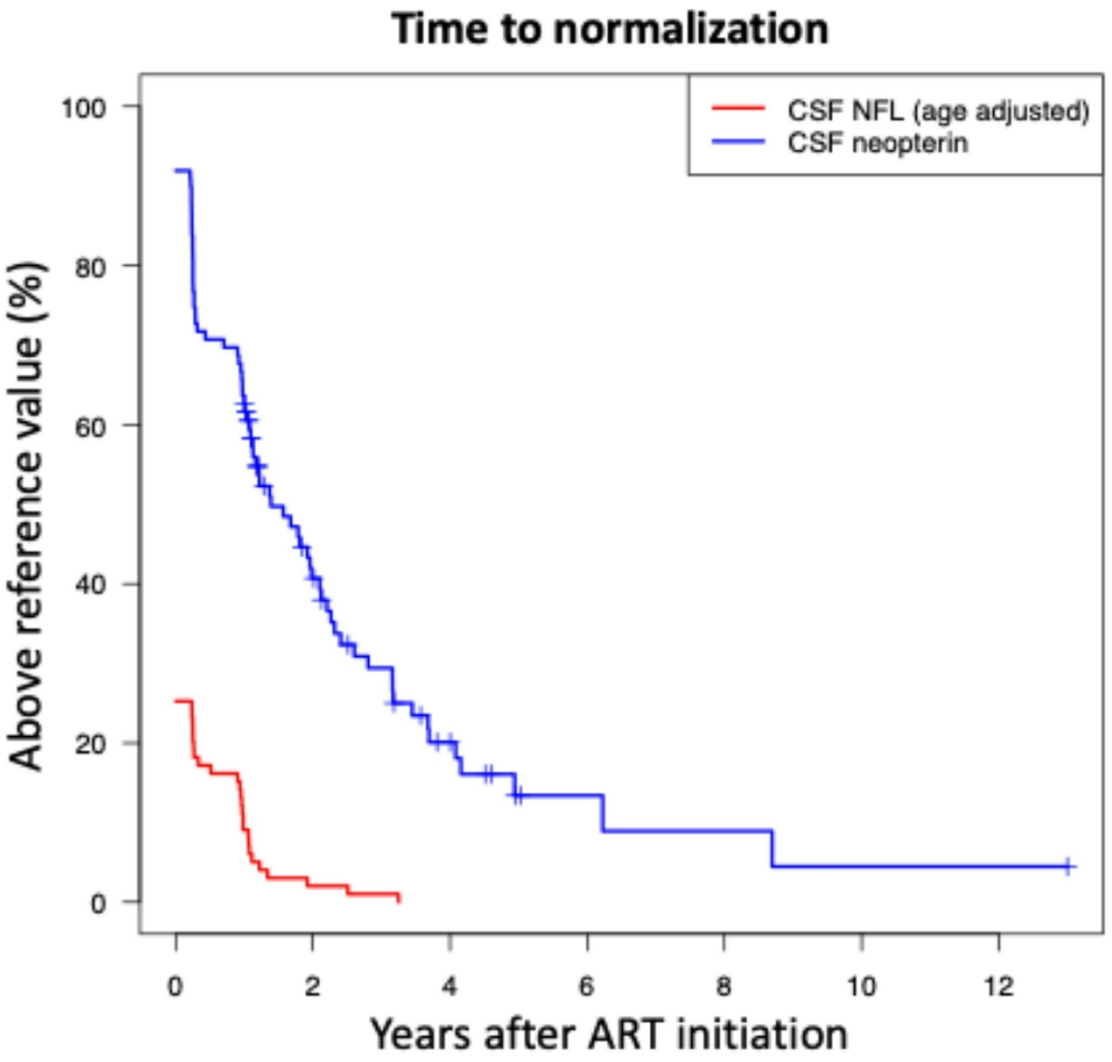
Kaplan-Meyer of time to normalization of cerebrospinal fluid NfL levels (age adjusted to 50 years, upper reference limit 967 ng/L) compared to neopterin (upper reference limit 5.8 nmol/mL)

In the 81 participants who were followed for more than 12 months, there was no further decline in any of the biomarkers. There was a trend towards lower age adjusted NfL and neopterin, while the opposite pattern was noted for YKL-40, GFAP, and sTREM2 (Table 2; Fig. 2). After the first year of follow-up, biomarker levels slowly increased by 1–3% per year. When adjusting for age, biomarker levels remained stable (Table 4).

**Table 4:** Biomarker change per year after the first year, before and after adjustment for ageing. Calculated using linear mixed model (*n* = 81)

Biomarker	Increase/year (%)	*p*	Increase/year (%), adjusted for ageing	*p*
NfL	1.3 (0.2–2.4)	0.025	-2.2 (-3.4–-0.9)	0.001
NfL age adj	-1.7 (-2.8–-0.7)	0.0016	-2.2 (-3.4–-0.9)	0.001
YKL-40	2.5 (2.1–2.9)	< 0.001	0.4 (-0.3–1.0)	0.28
GFAP	2.7 (2.1–3.4)	< 0.001	-0.2 (-1.4–1.0)	0.71
sTREM2	1.4 (1.1–1.8)	< 0.001	-0.8 (-1.7–0.0)	0.057
Neopterin	0.6 (-0.3–1.4)	0.18	-0.5 (-1,5–0.5)	0.30

Presented as % (95% CI)

NfL age adj: age adjusted to 50 years

Of the 25 participants with elevated NfL at baseline, levels normalized within four years of follow-up, with the large majority normalizing within 12 to 15 months. Eighteen participants in this subgroup were sampled at 3 months: 7 had CSF NfL within reference values by that timepoint. At 15 months, only 3 remained elevated above reference limit. By comparison, 91 participants (92%) had neopterin levels above normal reference limit at baseline, and 56 (57%) were still elevated at 12 months Fig. 2.

**Fig. 3:**
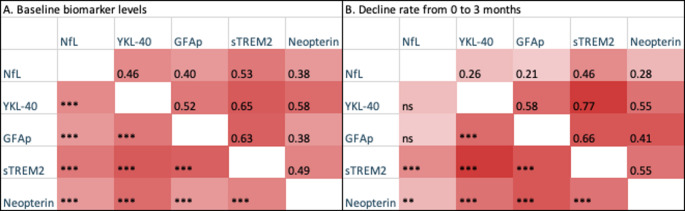
Heatmap of correlations of: (A) Cerebrospinal fluid biomarker levels at baseline, and (B) Decline rate from baseline to 3 months, calculated using partial correlations, adjusting for age. Upper right: r values. Lower left: significance level

There was a significant correlation between all biomarkers at baseline. The initial decline rate of NfL correlated significantly with the decline rates of sTREM and neopterin. At 12 months, similar correlations were observed, but the decline rate of NfL was also correlated with that of YKL-40 and GFAP. Baseline and initial decline rate correlations are presented in Fig. 3.

## Discussion

In this study, we investigated longitudinal changes in CSF biomarkers following ART initiation in neuroasymptomatic PWH. Participants with elevated baseline NfL showed pronounced declines in both NfL and inflammatory markers, whereas those with normal baseline NfL demonstrated reductions only in inflammatory markers. The steepest declines occurred during the first three months, with stabilization after one year when accounting for ageing.

Our findings are consistent with smaller earlier studies reporting reductions in NfL after ART initiation primarily in those with elevated baseline levels, though our cohort was larger and had longer follow-up. Our group has previously published two smaller studies, one of which longitudinally examined 78 neuroasymptomatic individuals (Krut et al. 2014) and one included 44 neuroasymptomatic PWH alongside individuals with HAD (Mellgren et al. 2007). Reduction of NfL after a median of 93–105 days of follow-up was only noted in participants with elevated baseline levels. Of those participants, 41 were also included in the present study. This cohort was larger with a lower incidence of elevated NfL at baseline (25% vs. 33–39%), and a higher median baseline CD4^+^ T-cell count (260 compared to 138–190 cells/µL. Another group found similar results in 31 PWH followed for a median of 200 days (Anderson et al. 2020). Two studies of plasma NfL, including 116 and 11 participants, have shown similar trajectories, with rapid initial declines followed by slower reductions and eventual stabilization, indicating parallel dynamics between CSF and blood NfL (Anderson et al. 2018; Ripamonti et al. 2022). After approximately one year, levels are stabilized in the large majority and thereafter slowly increase, as expected with age.

The decrease in inflammatory markers was similarly more pronounced in PWH with elevated baseline levels of CSF NfL, and followed the same biphasic pattern. A significant decrease in all inflammatory biomarkers was observed in the whole cohort and the NfL-“high” group after three and twelve months, while YKL-40, sTREM2, and neopterin decreased significantly also in the NfL-“normal” group. The decay of neopterin, which was by far the most pronounced, has been thoroughly described in relation to ART initiation (Yilmaz et al. 2013) and our results align with previous data. One study has measured YKL-40 after treatment initiation in 10 individuals with chronic and 24 individuals with acute HIV and found a 10% decrease after one year in chronic HIV and 18% after 6 months in acute HIV (Peluso et al. 2017). As a comparison, the average decrease of YKL-40 during the first year after ART in our cohort was 43%. Possible explanations of the different findings are our larger cohort and the higher median baseline NfL (586 vs. 327 ng/l), indicating a more pronounced CNS affection at baseline. For GFAP, only a single case report has documented normalization after 15 months of ART in a patient with HAD (Andersson et al. 2006). For sTREM2, no longitudinal data are currently available.

Taken together, the results suggest that in a subgroup of neuroasymptomatic PWH, both neuroinflammation and neuronal injury are present and can be effectively reduced by ART. This group is in a later phase of the infection, with lower CD4^+^ T-cells and higher plasma viral loads. The percentage of patients with elevated baseline biomarker levels resembles the incidence of HAD development in untreated HIV and an increase in NfL levels has been shown to precede the development of dementia (Gisslén et al. 2007). Theoretically, this subgroup might be the ones at the most risk of dementia if left untreated. It would be of interest to further investigate why some, but not all, develop more pronounced CNS engagement by HIV and what factors besides duration of infection and CD4^+^ T-cell depletion affect the vulnerability of the CNS, for example virus tropism and host factors. In PWH without signs of neuronal injury, there are still signs of intrathecal immune activation, with decreasing levels of YKL-40, sTREM2, and neopterin after treatment initiation.

The correlations between NfL and inflammatory biomarkers at baseline and during follow-up, and of the decline rates, underscore the link between neuroinflammation and axonal injury in HIV. Particularly notable was the strong association between YKL-40 and sTREM2, markers of astrocytic and microglial activation, respectively, supporting the interplay between these cell types in HIV neuropathogenesis. It has been shown that microglial activation can trigger neurotoxic reactive astrocytes by secretion of cytokines (Liddelow et al. 2017) and a correlation between YKL-40 and sTREM2 has also been found in Alzheimer’s disease (Heslegrave et al. 2016), normal pressure hydrocephalus (Yang et al. 2023), and prion disease (Diaz-Lucena et al. 2021). All biomarkers correlated to age, indicating that the ageing brain either is more prone to low grade inflammation or more vulnerable to the inflammatory mechanisms caused by HIV. Age correlation of NfL levels is well established (Yilmaz et al. 2017), and levels of CSF and plasma YKL-40 (Bojesen et al. 2011; Sutphen et al. 2015), CSF sTREM2 (Wang et al. 2024), and CSF GFAP (Vågberg et al. 2015) have also been reported to increase with age. The correlation to age explains why the biomarkers gradually start to increase with time after the initial period of decrease following ART initiation. To account for the age-related annual increase in NfL, we used age-adjusted NfL values in most analyses and correlation analysis was performed controlling for age.

Importantly, CSF HIV RNA did not correlate with NfL (*r* = 0.11, *p* = 0.27). Instead, correlations were found between CSF HIV RNA and baseline levels of YKL-40 and neopterin, which is expected when the virus primarily activates microglial and astroglial cells and the resulting inflammation, rather than direct viral effects, contributes to neuronal injury. Similar results were found in a previous report regarding untreated PWH(Hagberg et al. 2000) and asymptomatic CSF escape(Ulfhammer et al. 2025) while a correlation was reported between CSF HIV RNA and NfL in a study of 13 persons with primary HIV infection (Calcagno et al. 2024).

During follow-up beyond one year, biomarker levels gradually increased, except for neopterin, consistent with age-related changes. After adjusting for age, inflammatory biomarkers remained stable, while NfL showed a small annual decline, suggesting that some reduction of neuronal injury may persist for several years after ART initiation. Neopterin, however, demonstrated slower resolution: levels were above reference in 92% at baseline, 57% at one year, and 20% after four years, reflecting prolonged intrathecal immune activation, as also described by Yilmaz et al. (Yilmaz et al. 2013). The remaining immune activation is markedly reduced, likely to a level which does not cause detectable axonal injury as reflected by NfL, as observed in the subgroup with normal baseline NfL levels, where inflammatory biomarkers but not NfL declined after ART initiation.

In five participants, unexpected NfL elevations were observed shortly after ART initiation. One of them developed immune reconstitution inflammatory syndrome (IRIS) due to disseminated mycobacterium avium infection. Even though CNS IRIS due to MAC infection is relatively common (Kishida and Ajisawa 2008; Lee et al. 2013), this patient had no known CNS involvement of the MAC infection and no CNS symptoms. Another patient experienced an exacerbation of cutaneous vasculitis after ART initiation. One individual developed discrete cognitive impairment, which was not noted at inclusion but improved with continued ART. A fourth participant developed lumbago/ischias. In the fifth case, no plausible explanation for the NfL elevation was identified.

Ten participants developed later NfL spikes during ongoing ART. Three of these developed herpes zoster, one with documented secondary CSF escape. Herpes zoster is a recognized cause of inflammation, pleocytosis, and secondary HIV CSF escape, previously described by our group (Hagberg et al. 2020). Another participant was investigated for fever and neuropathic symptoms in the extremities without a clear etiology; symptoms partly resolved, but follow-up lumbar puncture was declined. One case of increased NfL followed a mycoplasma pneumonia without neurological complications. In the remaining five, no neurological or systemic symptoms were reported, leaving the cause of NfL elevation unexplained. Since longitudinal CSF sampling in healthy individuals is rare, and most available data are cross-sectional, the natural variability of NfL is uncertain. Transient elevations may occur after febrile illnesses or other CNS stressors. A minor ischemic event could also be considered, although no such symptoms or cardiovascular disease were reported in these cases.

The main limitations of this study include the absence of HIV-negative controls and lack of cognitive testing, which prevented assessment of subclinical improvement. Strengths include the relatively large sample size, extended follow-up, and comprehensive biomarker panel, which together provide robust insight into the CNS effects of ART initiation.

## Conclusion

The observed decreases in CSF NfL and biomarkers of immune activation imply that ART significantly reduces axonal injury and neuroinflammation within one year of treatment initiation, even in neuroasymptomatic individuals. Most of the decline in both NfL and inflammatory biomarkers occurred during the first three months and was most pronounced in participants with elevated NfL levels at baseline.

## Data Availability

The dataset supporting the conclusions of this article is available upon reasonable request to the corresponding author (linn.renborg@gu.se).

## Abbreviations

ART: antiretroviral therapy
CNS: central nervous system
CSF: cerebrospinal fluid
GFAP: glial fibrillary acidic protein
HAD: HIV associated dementia
NfL: neurofilament light protein
PWH: people with HIV
sTREM2: soluble triggering receptor expressed on myeloid cells 2
YKL-40: chitinase-3-like protein 1 [CHI3L1
